# Combination of urinary fibrinogen β-chain and tyrosine-phosphorylated proteins for the detection of bladder cancer

**DOI:** 10.2144/fsoa-2021-0060

**Published:** 2021-10-11

**Authors:** Giuliana Giribaldi, Claudia Filippini, Clara Viberti, Amina Khadjavi, Nicole Finesso, Daniela Ulliers, Stefano Turini, Bruno Emilio Bressan, Francesca Pecoraro, Mauro Prato, Alessandra Allione, Matteo De Bellis, Gabriele Montefusco, Giulia Bonomessi, Marco Allasia, Giuseppe Matullo, Francesco Soria, Paolo Gontero

**Affiliations:** 1 Department of Oncology, University of Turin, Turin, Italy; 2 Department of Surgical Sciences, University of Turin, Turin, Italy; 3 Department of Medical Sciences, University of Turin, Turin, Italy; 4 Department of Neurosciences, University of Turin, Turin, Italy; 5 Division of Urology, Department of Surgical Sciences, AOU Città della Salute e della Scienza, Torino School of Medicine, Turin, Italy; 6 SC Medical Genetics, AOU Città della Salute e della Scienza, Turin, Italy

**Keywords:** bladder cancer, fibrinogen β-chain, tyrosine-phosphorylated proteins, urinary markers

## Abstract

**Aim:**

To evaluate the performance of urinary fibrinogen β-chain (FBC) – either alone or associated with urinary tyrosine-phosphorylated proteins (UPY) – as bladder cancer (BCa) diagnostic biomarker.

**Materials & methods:**

164 subjects were tested.

**Results:**

Significantly different FBC and UPY levels were found between BCa patients and controls, as well as between low-grade and high-grade cancers. The diagnostic accuracy was 0.84 for FBC and 0.87 for UPY. The combination of FBC and UPY improved the accuracy to 0.91. The addition of clinical variables (age, gender, and smoking habit) to FBC and UPY into a model for BCa prediction significantly improved the accuracy to 0.99. The combination of FBC and UPY adjusted for clinical variables associates with the highest sensitivity and good specificity.

**Conclusion:**

Urinary FBC and UPY could be used as biomarkers for BCa diagnosis.

## Lay abstract

This research has developed and validated a highly accurate predictive model for BCa diagnosis based on the combination of two urinary biomarkers, fibrinogen β-chain (FBC), and urinary tyrosine-phosphorylated proteins (UPY), and some clinical variables (age, gender and smoking habit). If the preliminary promising results will be confirmed by external validations and prospective trials in selected clinical scenarios, our model could be transferred to clinical practice for screening of high-risk population.

## Graphical abstract

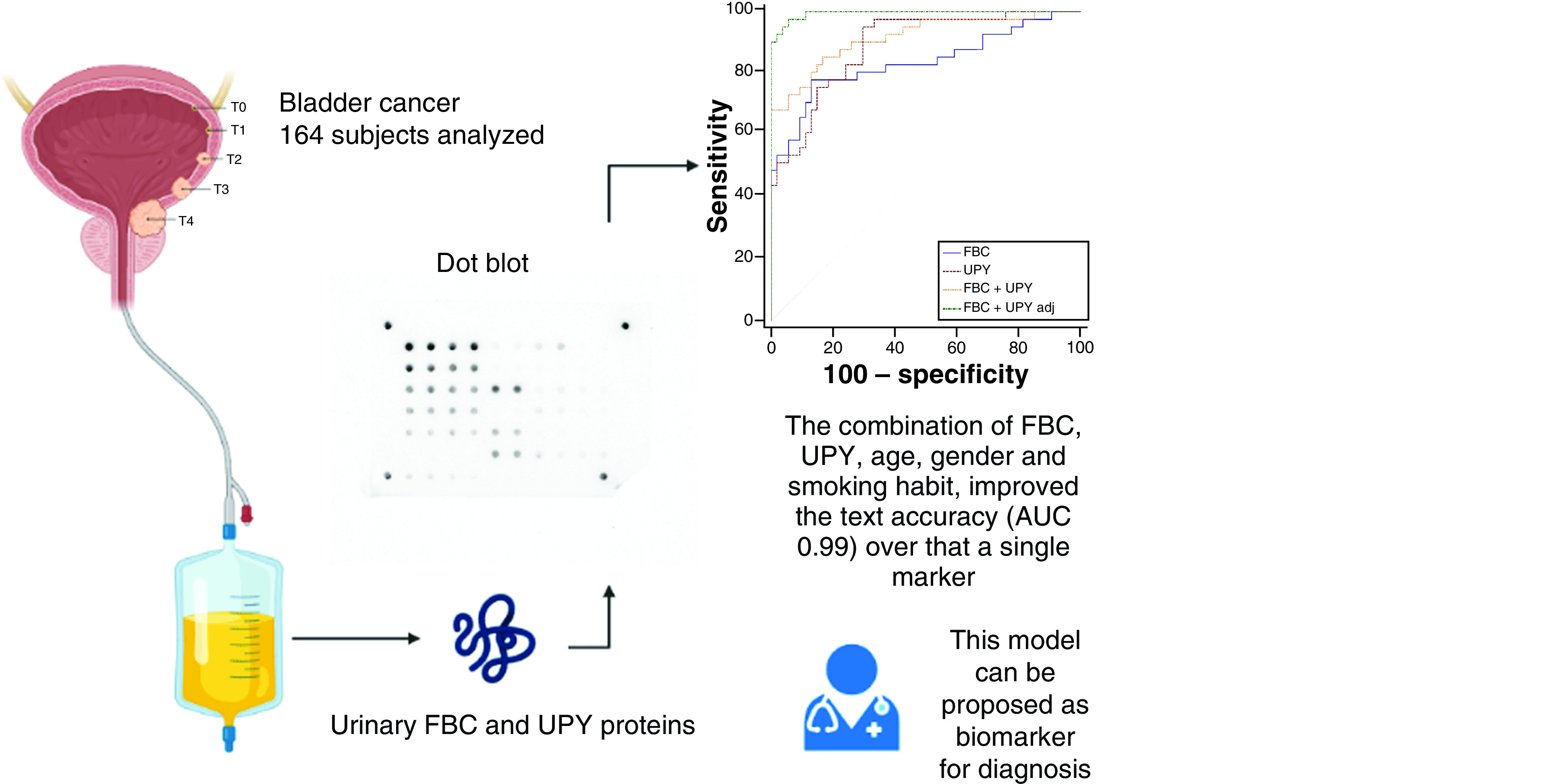

Bladder cancer (BCa) is a heterogeneous disease, thus representing a challenge for clinicians from the time of diagnosis to preventing recurrences and potentially death from the disease. At this time, BCa diagnostic algorithm is still based on invasive procedures such as cystoscopy and bladder biopsy/transurethral resection of the tumor [1]. In the diagnostic setting, molecular markers may help to identify the disease earlier, with potential impact on long-term oncologic outcomes, and may reduce the invasiveness and costs related to the endoscopic evaluation. So far, no molecular markers have clearly demonstrated to outperform the accuracy of urinary cytology for the detection of BCa and, therefore, none of them has been implemented in clinical practice. Despite some urinary markers have demonstrated to provide a better sensitivity compared with cytology, their specificity remains mainly unsatisfactory. This is of utmost importance since, if a marker should be used for diagnosis or screening purposes, it should have a high specificity to avoid a high number of unnecessary workups of healthy individuals, and a high positive predictive value (PPV) [2]. However, where the prevalence of the disease is low such as for BCa in the general population, and even in high-risk populations (i.e., heavy smokers or patients with microhematuria), also a highly sensitive and specific marker can have a low PPV [3]. Previously, our group demonstrated that levels of urinary tyrosine-phosphorylated proteins (UPY) may help to differentiate BCa patients from healthy controls [4]. Moreover, data obtained in this previous study strongly suggested a correlation between UPY levels and BCa stage and grade, thus representing a direct expression of tumor aggressiveness [5]. Historical studies have shown that elevated urinary fibrin/fibrinogen degradation product levels are associated with the presence of BCa [6–8]. More recently, elevated levels of urinary fibrinogen β-chain (FBC) have been correlated with higher tumor stage in patients with BCa [9]. The expression of fibrinogen has been shown to increase the growth and metastatic potential of lung cancer and melanoma cell lines [10] thus representing a indirect expression of tumor progression. Based on these previous findings, this study aimed at evaluating the performance of urinary FBC and the association of urinary FBC and UPY as diagnostic biomarkers for BCa. We hypothesized that UPY and FBC may be elevated in patients with BCa compared with healthy individuals, and that the association of the two markers may improve the diagnostic accuracy compared with UPY or FBC alone.

## Patients & methods

### Patients & sample collection

Urine samples from BCa patients were collected at the Division of Urology of AOU Città della Salute e della Scienza (Turin, Italy). Urine samples from healthy volunteers were obtained from the Blood Bank of the same institution. The cohort recruited patients with suspected BCa who were enrolled in this study before undergoing TUR (patients with a histological diagnosis different from BCa or with a previous BCa history were subsequently excluded) and as controls, people without symptoms or signs of BCa and without previous BCa history. All recruited subjects lived in the study area (for patients at the time of diagnosis); were over age of 18 years; were able to provide interview data and to sign an informed consent.

The subjects were classified as ‘current smokers’, ‘former smokers’ (quit smoking for at least 10 years before the inclusion) and ‘nonsmokers’, based on their attitude. Tumor grade was determined according to the 2004 WHO system. Voided urine samples (10–50 ml) were collected from the second micturition of the morning. Samples were stored at -80°C within 2 h from collection.

### Measurement of urinary tyrosine-phosphorylated proteins

**Figure 1.**
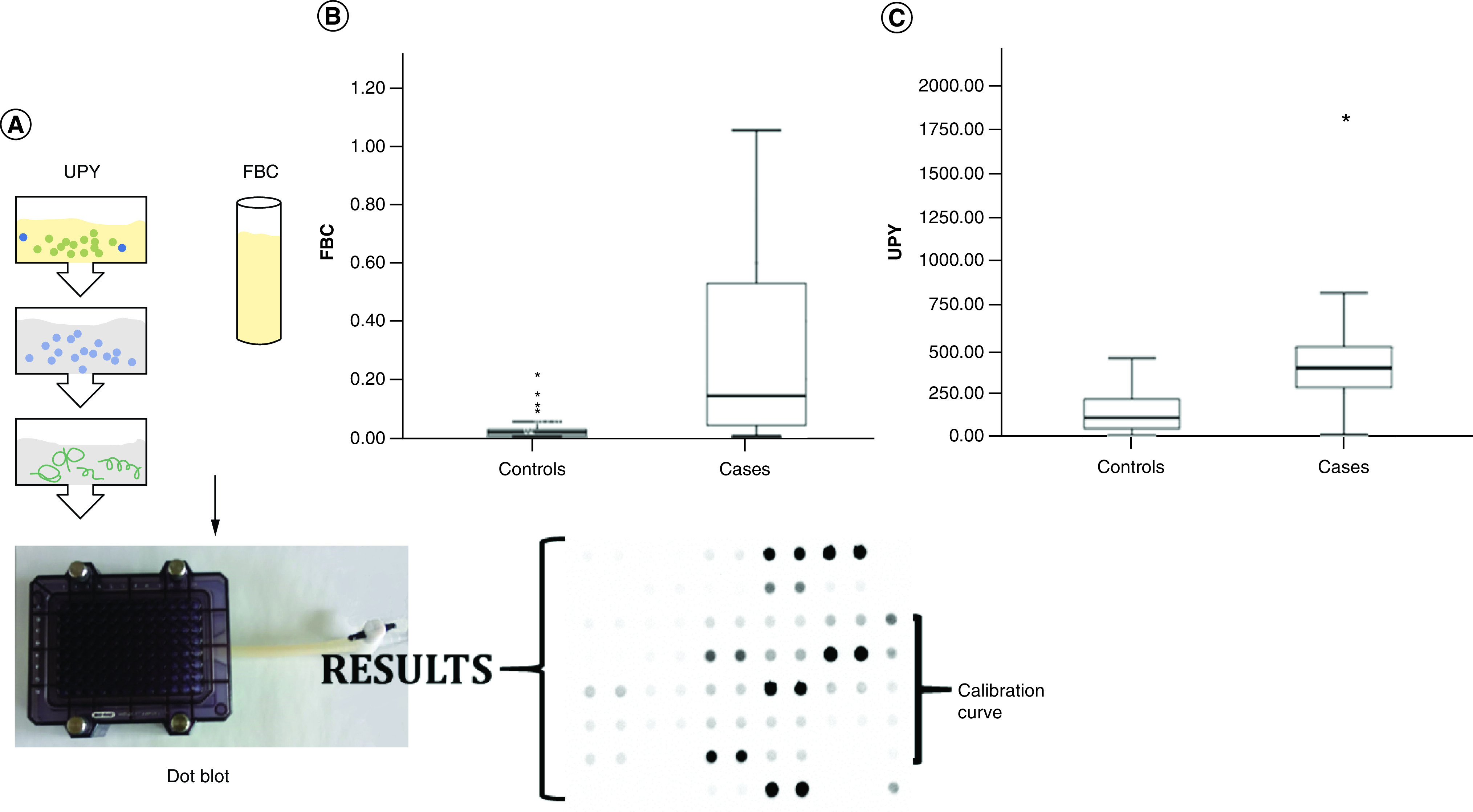
Urinary fibrinogen β-chain and tyrosine-phosphorylated protein levels in healthy subjects compared with bladder cancer patients. **(A)** Illustration of UPY and FBC detection methods. UPY levels were detected after a pre-analytical procedure. Briefly, IMAC-Select affinity gel (Sigma-Aldrich) was charged with CuSO_4_ for UPY enrichment. IMAC charged resin and 0.5 ml of urine were added to the SpinX centrifuge tube (Corning). The unbound fraction was discarded and UPY were eluted (for more detailed information see reference [4]). FBC levels were detected directly in urine supernatant without a pre-analytical procedure. Four microliters of supernatant from each patient for FBC detection or 200 μl of enriched UPY were spotted onto a prewet Hybond™ ECL nitrocellulose (Sigma-Aldrich) and placed in the Bio-Dot apparatus (Bio-Rad). The immunoreactivity was detected by chemiluminescence and densitometric analysis. **(B)** Analysis of FBC levels in samples of healthy subjects (controls) (n = 86) and BCa patients (n = 78): mean ± SD 0.025 ± 0.032 μg/μl versus 0.35 ± 0.45 μg/μl, respectively (Wilcoxon rank-sum test: p < 0.0001). **(C)** Analysis of UPY levels in samples of healthy subjects (n = 86) and BCa patients (n = 78): mean ± SD 136.88 ± 114.60 SU versus 415.89 ± 263.85 SU, respectively (Wilcoxon rank-sum test: p < 0.0001). BCa: Bladder cancer; FBC: Fibrinogen β-chain; IMAC: Immobilized metal affinity chromatography; SD: Standard deviation; SU: Standard unit; UPY: Urinary tyrosine-phosphorylated protein.

Urine samples were thawed and centrifuged for 20 min at 700 × g at 10°C, and supernatants were collected. Five hundred μl of supernatant from each patient were processed using the UPY-Assay for the evaluation of UPY levels (see Figure 1A) as already described [4]. Immunoreactivity was detected by a Chemidoc XRS+ System (Bio-Rad, CA, USA). A densitometric analysis was performed by the dot blot analyzer of ImageJ software (version 1.44). Using an external peptide calibration curve, UPY levels were interpolated and expressed as standard units (SU).

### Measurement of urinary fibrinogen β-chain

Urine samples were thawed and centrifuged for 20 min at 700 × g at 10°C and supernatants were collected. Four ml of supernatant from each patient were spotted onto a prewet Hybond™ ECL (Sigma-Aldrich, MO, USA) nitrocellulose placed in the Bio-Dot apparatus (Bio-Rad [see Figure 1A]). The nitrocellulose membrane was probed with anti-FBC antibody (Sigma-Aldrich) diluted 1:2000 and with anti-rabbit IgG secondary antibodies conjugated with Horseradish Peroxidase (GE Healthcare, IL, USA) diluted 1:7500. The membrane was washed three-times and immunoreactivity was detected by a Chemidoc XRS+ System (Bio-Rad). A densitometric analysis was performed by the dot blot analyzer of ImageJ software (version 1.44). Using FBC protein (Sigma-Aldrich) as calibration curve, FBC levels were interpolated and expressed as μg/μl of urine.

### Statistical analyses

Summary data are presented as means and standard deviations for continuous variables and as percentages for categorical variables. In univariate analysis continuous variables were compared with the use of the unpaired *t*-test or Mann–Whitney–Wilcoxon, according to distribution type. Categorical variables were compared with the use of the Chi-square test or Fisher exact test, when appropriate.

To evaluate relationships between FBC and UPY markers we calculated Spearman correlation coefficient. Patients were randomly divided into two independent sets: 98 subjects constituted the training set, whereas 66 subjects constituted the validation set. In training set we perform receiver-operating characteristics curve for FBC and UPY to assess the ability to discriminate BCa cases from healthy controls. Results are given as area under the curve (AUC) and CI at 95% level. Furthermore, the selected threshold values were those that maximized sensitivity (minimizing false negative classifications) and specificity (minimizing false positive classifications) according to Youden index J criterion. Positive and negative likelihood ratios were calculated. Multivariable analyses were preformed to evaluate the diagnostic power of investigated markers: two different logistic regression models were built combining FBC and UPY markers and adjusting for age, gender and smoking status. AUCs were compared with DeLong's test. In validation set standard formulae were used to calculate sensitivity and specificity. All tests were two tailed and the level of statistical significance was set at 0.05. All analyzed data have been deposited in an open repository (https://zenodo.org/).

## Results

### Urinary fibrinogen β-chain & tyrosine-phosphorylated protein levels in bladder cancer patients compared with healthy subjects

To compare the results obtained by analyzing FBC as a tumor marker in urine of BCa patients with data concerning UPY levels, a cohort of 164 samples was randomly selected from the 262 samples already analyzed for UPY in the previously published validation study [5]. Samples collected from 78 patients with BCa and 86 healthy subjects were therefore tested for both FBC and UPY. The demographic and clinical characteristics of enrolled subjects are shown in Table 1 & Supplementary Table 1.

**Table 1.** Participant demographics.

	All (n = 164)	Cases (n = 78)	Controls (n = 86)	p-value
Gender (male), n (%)	142 (87)	74 (95)	68 (80)	0.0046
Age (years), mean (SD)	62 (9.7)	66 (11.6)	58 (4.7)	<0.0001
Smoking^†^, n (%) Current smokers Nonsmokers Former smokers	39 (24) 76 (47) 46 (29)	24 (32) 6 (8) 45 (60)	15 (17) 70 (81) 1 (1)	<0.0001

† Missing values: n = 3.

SD: Standard deviation.

Overall, we observed a significant positive correlation between the two markers (Spearman correlation: p = 8.033E^11^; r = 0.48); stratifying for disease status, the significant correlation was confirmed, but was negative when considering only the healthy group (BCa cases: Spearman correlation: p < 2.2E^-16^; r = 0.68. Healthy subjects: Spearman correlation: p = 1.5E^-3^; r = -0.34). FBC levels showed a significant difference (Wilcoxon rank-sum test: p < 0.001) between patients with BCa versus healthy subjects (mean ± standard deviation [SD]: 0.35 ± 0.45 μg/μl vs 0.025 ± 0.032 μg/μl, respectively). Similarly, UPY levels confirmed a significant difference (p < 0.001 Wilcoxon rank-sum test) between BCa patients and healthy subjects (mean ± SD: 415.89 ± 263.85 SU vs 136.88 ± 114.60 SU, respectively [Figure 1]). To evaluate possible interferences, we tested 29 urine samples from subjects suspected of having BCa but found not to have BCa to be used as ‘urological controls’ (patients with cystitis and variable levels of leukocyturia and hematuria). FBC levels showed a significant difference (Wilcoxon rank-sum test: p = 0.01 ) between healthy subjects and urological controls (mean ± SD: 0.025 ± 0.032 vs 0.013 ± 0.014 μg/μl, respectively) but levels of urological controls appear to be lower than levels of healthy subjects. UPY levels were not significantly different (Wilcoxon’s rank-sum test: p = 0.08) between healthy subjects and urological controls (mean ± SD: 136.88 ± 114.60 SU vs 93.80 ± 93.38 S [Supplementary Table 2]).

### Correlation between urinary fibrinogen β-chain & tyrosine-phosphorylated protein levels with bladder cancer grade

**Figure 2.**
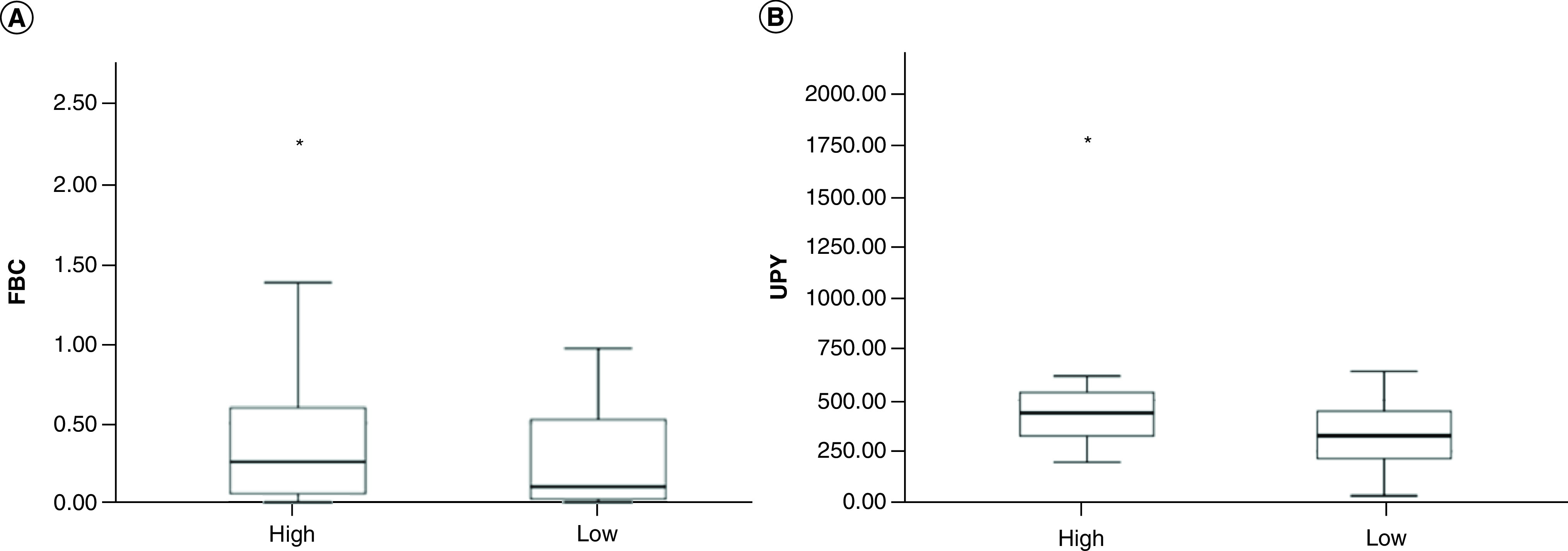
Urinary fibrinogen β-chain and tyrosine-phosphorylated protein levels in subjects classified according to bladder cancer WHO 2004 classification. **(A)** Analysis of FBC levels in patients with Low-grade BCa (n = 40) and with High-grade BCa (n = 35), mean ± SD 0.28 ± 0.38 μg/μl versus 0.45 ± 0.51 μg/μl (p = 0.04, Wilcoxon rank sum test). **(B)** Analysis of UPY levels in patients with Low-grade BCa (n = 40) and with High-grade BCa (n = 35), mean ± SD 351.44 ± 197.13 SU versus 502.66 ± 312.04 SU (p = 0.009, Wilcoxon rank sum test). BCa: Bladder cancer; FBC: Fibrinogen β-chain; SD: Standard deviation; UPY: Urinary tyrosine-phosphorylated protein.

BCa patients were stratified according to the WHO 2004 classification into high-grade (HG: n = 35) and low-grade (LG: n = 40) cancers (missing values: n = 3). A statistically significant different expression of urinary FBC and UPY between LG and HG subjects was observed, as shown in Figure 2. In details, FBC showed a mean ± SD level of 0.28 ± 0.38 μg/μl in LG tumors and 0.45 ± 0.51- μg/μl in HG tumors (Wilcoxon rank-sum test: p = 0.04); UPY showed a mean ± SD level of 351.44 ± 197.13 SU in LG tumors and 502.66 ± 312.04 SU in the HG tumors (Wilcoxon rank-sum test: p = 0.009). A significant positive correlation between FBC and UPY both in LG and HG tumor groups was observed (LG group: Spearman correlation: p = 1.3E^-4^; r = 0.48. HG group: Spearman correlation: p = 1.17E^-6^; r = 0.74).

### Accuracy of urinary fibrinogen β-chain & tyrosine-phosphorylated protein alone or in combination for the diagnosis of bladder cancer

**Figure 3.**
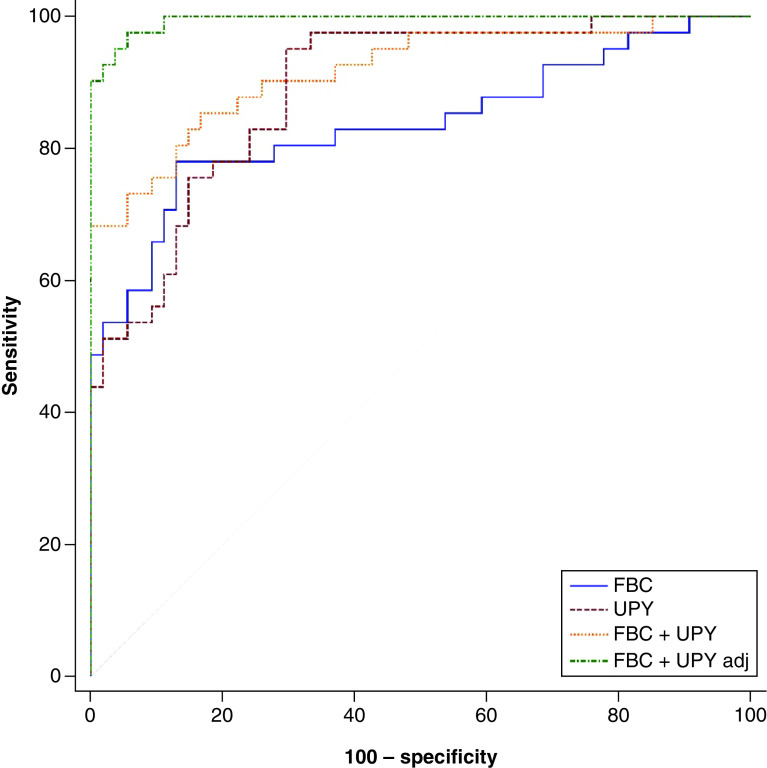
Receiver-operating characteristics curve analysis for discrimination of bladder cancer patients. The plot shows the ROC curve of the markers in the training set. FBC investigated the risk reached by FBC (AUC = 0.84; 95% CI: 0.76–0.91). UPY investigated the risk reached by UPY (AUC = 0.87; 95% CI: 0.79–0.93). FBC + UPY investigated the risk reached by the association of the two markers (AUC = 0.91; 95% CI: 0.83–0.96). FBC + UPY Adj investigated the risk reached by the association of the two markers adjusted for age, gender, and smoking habit (AUC = 0.99; 95% CI: 0.95–1.0). adj: Adjusted; AUC: Area under the curve; FBC: Fibrinogen β-chain; ROC: Receiver-operating characteristics; UPY: Urinary tyrosine-phosphorylated protein.

To assess the predictive accuracy of urinary FBC and UPY levels for the diagnosis of BCa, a ROC curve analysis in the training set of subjects (n = 98) was performed. Using only the markers as BCa predictors, we obtained an AUC of 0.84 for FBC (95% CI: 0.76–0.91), and an AUC of 0.87 (95% CI: 0.79–0.93) for UPY (Figure 3). We obtained a sensitivity of 79.07% (95% CI: 64.0–90.0) and a specificity of 87.27% (95% CI: 75.5–94.7) with a PPV of 81.0% and a NPV of 83.9% for FBC and a sensitivity of 93.02% (95% CI: 80.9–98.5) and a specificity of 70.91% (95% CI: 57.1–82.48) with a PPV of 71.4% and a NPV of 92.9% for UPY (Table 2). A logistic regression analysis was then performed to evaluate whether a combination of the two markers could better discriminate between patients and healthy subjects. The combination of FBC and UPY improved the accuracy of the test (AUC = 0.91, 95% CI: 0.83–0.96) (Figure 3); the combination of the two markers displayed a sensitivity of 67.44% (95% CI: 51.5–80.9) and a specificity of 100.0% (95% CI: 93.5–100.0) with a PPV of 100% and a NPV of 79.7%. The addition of clinical variables (age, gender and smoking habit) to FBC and UPY into a model for the prediction of BCa (FBC + UPY adjusted [adj]) significantly improved its accuracy (AUC = 0.99; 95% CI: 0.95–1.0) (Figure 3). This model reached the highest sensitivity of 97.57% (95% CI: 87.1–99.9) and a good specificity of 94.44% (95% CI: 84.6–98.8), with a PPV of 93.0% and a NPV of 98.1% (Table 2). The discriminative power performances were evaluated by the DeLong’s test (model FBC vs model FBC + UPY adj: p = 0.0003; model UPY vs model FBC + UPY adjusted: p = 0.0008; and model FBC + UPY vs model FBC + UPY adj: p = 0.0051 [Figure 3]). Obtained data were subsequently validated in an independent set of 66 subjects (validation set). Using only the markers as BCa predictors, we obtained a sensitivity of 73.33% (95% CI: 57.51–89.16) and a specificity of 80.56 % (95% CI: 67.63–93.48) with a PPV of 75.9% and a NPV of 78.4 % for FBC, and a sensitivity of 86.67% (95% CI: 80.9–98.5) and a specificity of 75.00% (95% CI: 60.86–89.14) with a PPV of 74.3 % and a NPV of 87.1% for UPY. The combination of FBC and UPY displayed a sensitivity of 63.33% (95% CI: 46.09–80.58) and a specificity of 94.44% (95% CI: 86.96–100.0), with a PPV of 90.5% and an NPV of 75.6%. The addition of clinical variables to FBC and UPY into a model for the prediction of BCa (FBC + UPY adj) led to a sensitivity of 90.00% (95% CI: 79.26–100.0) and a specificity of 91.67% (95% CI: 82.64–100.0) with a PPV of 90.00% and an NPV of 91.67% (Table 2). We can, therefore, conclude that FBC and UPY performed well in both the training and validation sets, thus confirming the reproducibility of the results.

**Table 2.** Performances of Fibrinogen β-chain, urinary tyrosine-phosphorylated protein, combination fibrinogen β-chain + urinary tyrosine-phosphorylated protein and combination fibrinogen β-chain + urinary tyrosine-phosphorylated protein adj (fibrinogen β-chain + urinary tyrosine-phosphorylated protein + age + gender + smoking habit) based on single-marker cut-off for the diagnosis of bladder cancer.

	Criterion	Sensitivity (95% CI)	Specificity (95% CI)	PPV	NPV
FBC					
Training set Validation set	>0.0398	79.07 (64.0–90.0) 73.33 (57.51–89.16)	87.27 (75.5–94.7) 80.56 (67.63–93.48)	81 75.9	83.9 78.4
UPY					
Training set Validation set	>189.8119	93.02 (80.9 98.5) 86.67 (74.50–98.83)	70.91 (57.1–82.48) 75.00 (60.86–89.14)	71.4 74.3	92.9 87.1
FBC + UPY					
Training set Validation set	>0.5991	67.44 (51.5–80.9) 63,33 (46.09–80.58)	100.00 (93.5–100.0) 94.44 (86.96–100.0)	100 90.5	79.7 75.6
FBC + UPY adj					
Training set Validation set	>0.3805	97.56 (87.1–99.9) 90.00 (79.26–100.0)	94.44 (84.6–98.8) 91.67 (82.64–100.0)	93 90	98.1 91.7

adj: Adjusted; FBC: Fibrinogen β-chain; NPV: Negative predictive value; PPV: Positive predictive value; UPY: Urinary tyrosine-phosphorylated protein.

## Discussion

In this retrospective single center study, we demonstrated and validated the utility of FBC and UPY as urinary biomarkers for BCa diagnosis and we developed a highly accurate predictive model for the prediction of BCa. We found that FBC may be used as urinary biomarker for BCa diagnosis. FBC has been reported to be involved in growth, proliferation and metastatic potential in cancer cell lines [10,11]. More than 40 years ago, Pineiro *et al.* firstly reported the value of urinary fibrinogen degradation products as possible biomarkers for BCa [6]. Subsequently, the FBC expression in BCa tissue was demonstrated, and FBC levels correlated with higher tumor stages [9], while a proteomic analysis of urinary biomarker candidates pointed out FBC and α-1-antitrypsin as most interesting biomarkers for BCa [12]. A high level of fibrinogen and D-dimer has recently been demonstrated in urological tumors not only in the case of invasive muscle tumors but also in the plasma of patients with localized tumors [13]. Furthermore, an increase of coagulation factors (D-dimers, von Willebrand Factor, thrombin, fibrin-/ogen, soluble P-selectin and prothrombin fragments 1 + 2) in genitourinary cancers has been associated with vascular thrombosis and tumor initiation, as well as metastatic progression by John *et al.* [14]; indeed the deposition of fibrin-/ogen and other adhesive glycoproteins in the cellular matrix together with the action of metalloproteinases [15] promotes adhesion, proliferation and migration both during angiogenesis and during tumor growth [16]. Of note preoperative elevated plasma fibrinogen level were considered as useful biomarkers as predictor of poor survival after radical nephroureterectomy and of worse pathological feature in upper tract urothelial carcinoma patients [17]. However, to date, its role as urinary biomarker for BCa diagnosis has not been further investigated. In our study, urinary FBC levels significantly differed between BCa patients and healthy controls and demonstrated to be able to predict the presence of BCa with an accuracy of 84%.

We confirmed the role of UPY as BCa biomarker. Our group previously demonstrated different UPY levels in 262 patients (92 BCa and 170 healthy controls) [5]: at the best cut-off value, the sensitivity of the UPY test was 80.43% and the specificity 78.82% for BCa diagnosis. In this study, the UPY test alone was able to detect BCa with an accuracy of 87%.

We showed that the combination of urinary FBC and UPY with standard clinical prognosticators such as age, gender and smoking habit in a model for the prediction of BCa reached the highest diagnostic accuracy, being able to discriminate BCa patients from healthy in more than nine patients every ten.

The search for accurate urinary biomarkers for BCa diagnosis is ongoing. To date, none of the proposed urinary biomarkers for the diagnosis of BCa have been integrated in current international guidelines and clinical practice, mainly due to their unsatisfactory performances and/or to the lack of prospective validations. Accurate urinary biomarkers for BCa diagnosis may be used for screening purposes, especially in high-risk populations such as heavy-smokers or workers with occupational purposes, in which the incidence of BCa is usually higher compared with the general population [2]. To date, the most-extensively investigated biomarkers in this context are urinary dipstick for hematuria and urinary cytology. Britton *et al.* evaluated the ability of urine dipsticks to detect early BCa in asymptomatic men, thus aiming to improve the long-term oncological outcomes [18]. Among 2356 males, 474 had a positive dipstick but only 17 were found to have BCa at subsequent cystoscopy. These initial unsatisfactory results were confirmed in a recently published Cochrane review [19]. So far, four urinary biomarkers have received the US FDA approval for the detection of BCa (immunocytology, Urovysion, NMP22 and BTA), with sensitivity and specificity rates ranging from 47 to 85% and from 53 to 95%, respectively [2]. To date, their use as a single marker in clinical practice remains limited.

Now, a single marker may not be able to provide sufficient performances to be used alone; conversely, the search is moving toward the development of predictive models containing a panel of biomarkers in addition to clinical variables [20]. A classic example is represented by the Cxbladder Monitor, a non-invasive urine test designed to rule out the presence of recurrent urothelial carcinoma and that combines gene expression, clinical and patient data [21]. To date, Cxbladder Monitor is one of the most promising urinary tests for BCa follow up (sensitivity 93%, NPV 97%), and its use in clinical practice has been recently approved in some countries (i.e., New Zealand). Based on these considerations, we developed and validated a highly accurate predictive model for BCa detection based on two urinary biomarkers and patient’s characteristics. The low concordance rate found between FBC and UPY in the training set probably due to their different biological significance (UPY index of tumor aggressiveness and FBC index of the patient’s coagulation status and of tumor progression) demonstrated the need to combine the two markers to reach the highest performances and to obtain a patient specific urinary marker ‘fingerprint’.

The results of our study may have several practical implications. If these promising results will be confirmed by external validations and prospective trials in selected clinical scenarios, our test may enter in clinical practice for the screening of high-risk populations. This may also be facilitated by the fact that our test, being based on the expression of urinary proteins, is low-cost (around €60) and easy-reproducible, thus being able to favorably impact on the healthcare costs related to the BCa detection algorithm (based on urinary cytology, cystoscopy and abdomen ultrasound/computed tomography). The ability of the test in discriminating between LG and HG tumors may be of use to predict BCa aggressiveness and, potentially, response to intravesical therapy [22]. In this regard, our group is now conducting a study to evaluate the ability of the test in combination with other urine biomarkers to predict response to intravesical Bacillus Calmette–Guerin in high-risk non-muscle invasive bladder cancer patients.

## Limitations

Our study is, of course, not devoid of limitations, mainly related to its retrospective nature and to the small sample size. We were not able to assess the presence and the effects of other important factors such as the presence of micro or gross hematuria on the accuracy of the test. Moreover, we were not able to evaluate the added impact of other important prognostic variables in a model for the prediction of BCa. There is evidence that the addiction of prognostic baseline variables such as BMI [23] and inflammation status (i.e., the Systemic combined inflammatory score [24] that combines the levels of serum inflammatory markers, those of CRP, the presence of hypoalbuminemia and the prognostic nutritional index) may improve the accuracy of the model. Finally, we were not able to compare the performances of our model to that of urinary cytology and, eventually, to add urinary cytology to the multivariable model.

## Conclusion

Urinary FBC and UPY can be used as biomarkers for BCa diagnosis. Their implementation into a model for the prediction of BCa (together with clinical variables such as age, gender and smoking habit) led to a diagnostic accuracy of 99%.

## Future perspective

Pending external validations and prospective analysis, this urinary test may be used in the next future for the screening of high-risk populations.

**Summary points**

- Bladder cancer (BCa) is a heterogeneous disease, thus representing a challenge for clinicians from the time of diagnosis to preventing recurrences and potentially death from the disease.
- BCa diagnostic algorithm is still based on invasive procedures such as cystoscopy and bladder biopsy/transurethral resection of the tumor.
- None of the proposed urinary biomarkers for the diagnosis of BCa have been integrated in current international guidelines and clinical practice.
- The search is moving toward the development of predictive models containing a panel of biomarkers in addition to clinical variables because a single marker may not be able to provide sufficient performances to be used alone.
- Previously, our group demonstrated that levels of urinary tyrosine-phosphorylated proteins (UPY) may help to differentiate BCa patients from healthy controls suggesting a correlation between UPY levels and BCa stage and grade.
- Elevated levels of urinary fibrinogen β-chain (FBC) have been correlated with higher tumor stage in patients with BCa.
- We have showed: levels of FBC may help to differentiate BCa patients from healthy controls suggesting a correlation between FBC levels and BCa stage and grade; the combination of urinary FBC and UPY with standard clinical prognosticators (age, gender and smoking habit) in a model for the prediction of BCa reached the highest diagnostic accuracy (area under the curve 0.99).
- If the preliminary promising results will be confirmed by external validations and prospective trials in selected clinical scenarios, our test may enter in clinical practice for the screening of high-risk populations.
