# Supplementary material for: Combination of urinary fibrinogen β-chain and tyrosine-phosphorylated proteins for the detection of bladder cancer
Source: Future Sci OA. 2021 Oct 11;7(9):FSO758. doi: 10.2144/fsoa-2021-0060 (PMC8558871; doi:10.2144/fsoa-2021-0060)
Supplement: Supplementary file 1 [file fsoa-07-758-s1.docx]

**Supplementary Materials:**

**Table S1**: Patient histology

| **Histology at 1^st^ diagnosis** *n (%)* | |
| --- | --- |
| CIS | 3 (4) |
| Ta | 48 (62) |
| T1 | 16 (21) |
| T2-3 | 10 (13) |
| **Grading at 1^st^ diagnosis** *n (%)* | |
| CIS | 3 (4) |
| 1 | 27 (35) |
| 2 | 26 (33) |
| 3 | 22 (28) |
| **WHO 2004** *n (%)* | |
| Low grade | 40 (51) |
| High grade | 35 (45) |
| Missing | 3 (4) |

**Table S2:** Performances of FBC and UPY in urological controls

|  | **Controls**  **N=115** | **FBC** (ng/µl)  (mean ± SD) | **UPY (**SU)  (mean ± SD) |
| --- | --- | --- | --- |
| **Urological controls,** *n (%)* | 20 (17.4) | 0.015 ± 0.017 | 101,3 ± 105,7 |
| **Urological controls with haematuria,** *n (%)* | 9 (7.8) | 0.008 ± 0.001 | 77 ± 59.4 |
| **Urological controls + controls with haematuria,** *n (%)* | 29 (25.2) | 0.013 ± 0.014 | 93.8 ± 93.4 |
| **Healthy controls** *n (%)* | 86 (74,8) | 0.025 ± 0.032 | 135.94 ± 114.26 |
